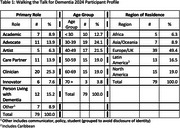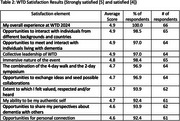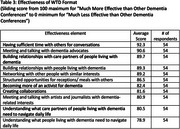# The Scope and Impact of Walking the Talk for Dementia 2024

**DOI:** 10.1002/alz70858_097738

**Published:** 2025-12-24

**Authors:** Fernando Aguzzoli Peres, Sherril B Gelmon, Walter D Dawson, Kate Irving, Lingani Mbakile‐Mahlanza, Jonathan Adrian Zegarra‐Valdivia, Iracema Leroi

**Affiliations:** ^1^ Global Brain Health Institute, Dublin, Dublin, Ireland; ^2^ Walking the Talk for Dementia, Porto Alegre, Rio Grande do Sul, Brazil; ^3^ OHSU‐PSU School of Public Health, Portland, OR, USA; ^4^ GBHI Lived Experience Panel, Portland, OR, USA; ^5^ Global Brain Health Institute, San Francisco, CA, USA; ^6^ Oregon Health & Science University, Portland, OR, USA; ^7^ Dublin City University, Dublin, Ireland; ^8^ University of Botswana, Gaborone, Botswana; ^9^ Achucarro Basque Center for Neuroscience, Leioa, Spain; ^10^ Global Brain Health Institute (GBHI), University of California San Francisco, San Francisco, CA, USA; ^11^ Universidad Señor de Sipán, Chiclayo, Peru; ^12^ Global Brain Health Institute, Dublin, Ireland

## Abstract

**Background:**

Walking the Talk for Dementia (WTD) is a global initiative designed to challenge the stigma surrounding dementia, foster intergenerational dialogue, and inspire a renewed sense of purpose among participants. The event combines a 4‐day, 40 km pilgrimage to Santiago de Compostela with a 2‐ day symposium, creating an immersive space to reflect on the physical, mental, spiritual, and societal dimensions of living with dementia. WTD prioritizes diverse, multicultural, and multistakeholder engagement, aiming to elevate the voices of people living with dementia, caregivers, and advocates while promoting inclusive and sustainable global solutions for brain health. This approach allowed the integration of professional and lived experiences, fostering a collaborative platform to address stigma, increase awareness, and build a community.

**Method:**

Data were collected before, during, and after the event through surveys and personal reflections from 79 participants representing 27 countries across five continents in various roles (Table 1).

**Results:**

Post‐event surveys demonstrated high levels of satisfaction with all elements (Table 2). Participants rated the effectiveness of the unique WTD format (Table 3), and felt that the experience was superior to many traditional academic conferences regarding networking and engaging with people with lived experience. Qualitative responses highlighted the event's transformative nature.

One care partner shared, “I feel more empowered and with a renewed sense of purpose in my role.” A person living with dementia stated, “I have found an even greater purpose on a global level to share my voice and expect to be heard. I am ready.” An academic reflected, “The experience deepened my empathy and motivated me to work better not just as a researcher, but also as an advocate for people living with dementia.”

**Conclusion:**

Walking the Talk for Dementia 2024 exemplifies how dementia changemakers across disciplines and regions can be inspired. By integrating physical activity, interpersonal dialogue, and potentially emotional experiences, WTD empowers participants to transform stigma into understanding, and challenges them to create opportunities for global advocacy. The voices of participants underscore its profound impact, showcasing WTD as a powerful catalyst for change in the dementia landscape.